# Treatment of Anemia of Chronic Disease with True Iron Deficiency in Pregnancy

**DOI:** 10.1155/2017/4265091

**Published:** 2017-12-04

**Authors:** Gabriela Amstad Bencaiova, Alexander Krafft, Roland Zimmermann, Tilo Burkhardt

**Affiliations:** ^1^Department of Obstetrics and Gynecology, University Hospital of Basel, Basel, Switzerland; ^2^Department of Obstetrics and Gynecology, University Hospital of Zurich, Zurich, Switzerland

## Abstract

**Objective:**

We assess and compare the efficacy of anemia treatment in pregnant women with anemia of chronic disease with true iron deficiency and in women with iron deficiency anemia.

**Study Design:**

Fifty patients with moderate anemia (hemoglobin 8.0–9.9 g/dl) and iron deficiency (ferritin < 15 *μ*g/l) were treated in the Anemia Clinic at the Department of Obstetrics.

**Results:**

All patients showed stimulation of erythropoiesis as evidenced by an increase in reticulocyte count at day eight of therapy and showed an increase in hemoglobin and hematocrit at the end of therapy (*p* < 0.001). The target hemoglobin (≥10.5 g/dl) was achieved in 45/50 women (90%). 12 patients showed anemia of chronic disease with true iron deficiency (12/50; 24%). Seven women (7/12; 59%) with anemia of chronic disease and iron deficiency responded well to anemia treatment. 50% of women with anemia of chronic disease and iron deficiency (3/6) responded well to intravenous iron, and 67% (4/6) responded well to the combination of intravenous iron and recombinant human erythropoietin.

**Conclusion:**

Because of frequent true iron deficiency in pregnant women with anemia of chronic disease, anemia of chronic disease in pregnancy is often falsely diagnosed as iron deficiency anemia.

## 1. Introduction

Anemia is one of the most common problems in pregnancy. Anemia diagnosed before mid-pregnancy contributes significantly to maternal and perinatal morbidity and mortality [1–3]. These include premature birth, low birth weight, intrauterine growth restriction, stillbirth, premature rupture of membranes, increased susceptibility to infection, and maternal postpartal complications such as breastfeeding problems, depression, and fatigue [1–5]. Placental development is also affected by anemia and hypoxia, causing abnormal trophoblast invasion and release of hypoxia inducible factor. This consequently increases the incidence of placenta previa and preterm placenta abruption. Mireku et al. report a link between mothers who had anemia lower than 9.0 g/dl during pregnancy with a lower cognitive and motor development in one-year-old children [6].

The etiology of gestational anemia may include the most common causes: deficiencies of iron, folate, vitamin B12, vitamin A, thalassemia, and so on. Iron deficiency anemia is the most common cause of anemia in pregnancy. The criteria of moderate iron deficiency anemia (IDA) are low hemoglobin levels (hemoglobin between 8.0 and 9.9 g/dl) and depleted iron stores (ferritin < 15 *μ*g/l) [1]. A prevalence of iron deficiency anemia in different regions of the world has varied from 12 to 43% [1, 7, 8]. Anemia of chronic disorders (ACD) also termed as anemia of chronic disease or anemia of chronic inflammation is considered to be the second most frequent anemia in the world and is primarily found in subjects suffering from disorders that evolve from the activation of the immune system [9, 10]. The measurements of hypochromic red blood cells (HRC), the reticulocyte hemoglobin content (CHr), and red blood cell distribution width (RDW) provide an accurate description of hemoglobinization of red blood cells and reticulocytes [11–15]. Because of the long circulating lifespan of mature erythrocytes, HRC values mainly provide information of the iron status during the last four months [11]. Hypochromic red blood cells show lower erythrocyte deformability and shortened lifespan so increased levels of HRC cause aggravation of anemia [16]. Consequently, the reduction of hypochromic red blood cells plays an important role in the effective correction of anemia. On the other hand as reticulocytes have a lifespan of one to two days in circulation, changes in the CHr identify variations in iron demand to bone marrow more rapidly. The determination of the percentage of hypochromic red cells or reticulocyte hemoglobin content can be useful in detecting accompanying iron restricted erythropoiesis in patients with anemia of chronic disease [10, 17]. In contrast to iron deficiency anemia, erythropoietin response in anemia of chronic disease is inadequate for the degree of anemia in most but not all conditions [10]. The difference between anemia of chronic disease and iron deficiency anemia thus relates to the latter as an absolute iron deficiency, whereas the pathophysiology of anemia of chronic disease is multifactorial and is characterized by a disturbance in iron utilization with normal iron stores [9, 10].

Isolated IDA can often be prevented by iron supplementation and, once it has manifested, is preferentially treated by oral iron salts. Parenteral iron treatment is an alternative to consider, especially when a rapid correction is needed, or gastrointestinal malabsorption or active inflammatory disease dampens dietary iron absorption in ACD [18, 19]. In patients with intolerance to oral iron therapy, parenteral iron is the treatment of choice. Many subjects with ACD who are under causative treatment for their underlying condition do not have an adequate hemoglobin (Hb) response to iron therapy. The erythropoietin (EPO) resistance of the erythron or renal EPO deficiency may be present, such that recombinant human erythropoietin (rhEPO) therapy should be considered as add-on therapy for anemia [9, 10].

In pregnant women with moderate and severe anemia, parenteral administration of iron potentially in combination with rhEPO can be an important alternative; it also provides quick and efficient correction of the total iron deficit because it not only corrects the anemia, but also builds up iron stores [20–25]. To our knowledge, there has been no study about the treatment of anemia of chronic disease with true iron deficiency (ID) in pregnancy. The aim of this study was to investigate the response to therapy in pregnant women fulfilling criteria for anemia of chronic disease and iron deficiency.

## 2. Material and Methods

The study was approved by the hospital's Ethics Committee, and consent was obtained from the patients. The study was registered at https://www.clinicaltrials.gov/ (NCT03317210). Primary endpoint was the assessment and comparison of the efficacy of anemia treatment in pregnant women with iron deficiency anemia and anemia of chronic disease with true iron deficiency. Secondary endpoint was the target hemoglobin ≥10.5 g/dl.

### 2.1. Study Population and Criteria

Fifty anemic pregnant women with moderate anemia were prospectively observed and treated in the Anemia Clinic at the Department of Obstetrics, University Hospital Zurich. All patients had singleton pregnancies. All pregnant women fulfilled criteria of moderate iron deficiency anemia defined as hemoglobin between 8.0 and 9.9 g/dl and serum ferritin <15 *μ*g/l. In all women, the analyses of a blood count, iron status, erythropoietin, cross reactive protein (CRP), folic acid, and vitamin B_12_ were conducted.

Exclusion criteria were anemia of other etiology (i.e., vitamin B_12_ deficiency, folic acid deficiency, hemoglobinopathy, etc.), liver or kidney disease, and multiples. Women with mean corpuscular hemoglobin (MCH) ≤25 pg, mean corpuscular volume (MCV) ≤75 fl, and percentage of microcytic erythrocytes (MRC) ≥3% were tested for hemoglobinopathies.

Flow cytometry was used to determine the total iron demand of erythropoiesis by measuring HRC, CHr, and RDW. Iron deficiency anemia is defined as anemia with depleted iron stores and with elevated levels of HRC >2.5%, decreased CHr <28 pg, and elevated RDW >15%. In combination with the indicators of erythropoiesis (hemoglobin, red blood cells, and hematocrit) as well as the indicators of iron household (serum ferritin and transferrin saturation), we identified anemia of chronic disease with reduced iron stores but with normal level of hypochromic erythrocytes (HRC < 2.5%), normal reticulocyte hemoglobin content (CHr >28 pg), normal red blood cell distribution width (RDW < 15%), and low serum EPO levels for the grade of anemia (serum EPO < 50 U/l by Hb < 10 g/dl).

### 2.2. Laboratory Assessment

Hb, red blood cells (RBC), hematocrit (HCT), MCV, MRC, HRC, RDW, and CHr were measured using an ADVIA® hematology analyser system (Bayer Diagnostics, Leverkusen, Germany). MCH was automatically calculated from Hb and RBC. Iron status was assessed by chemiluminescence-immunoassay (ACS 190; Ciba/Corning Diagnostic Corp., Cleveland, OH) of serum ferritin, iron, and transferrin. Transferrin saturation was calculated as 100 × iron/2 × transferrin. Radioimmunoassay was performed to determine vitamin B_12_, folic acid, and the levels of serum EPO. CRP was assessed through immunoturbidimetry. The hematological parameters were checked twice a week in the anemia clinic and iron status was checked once a week.

### 2.3. Therapy Protocol

**Figure 1:**
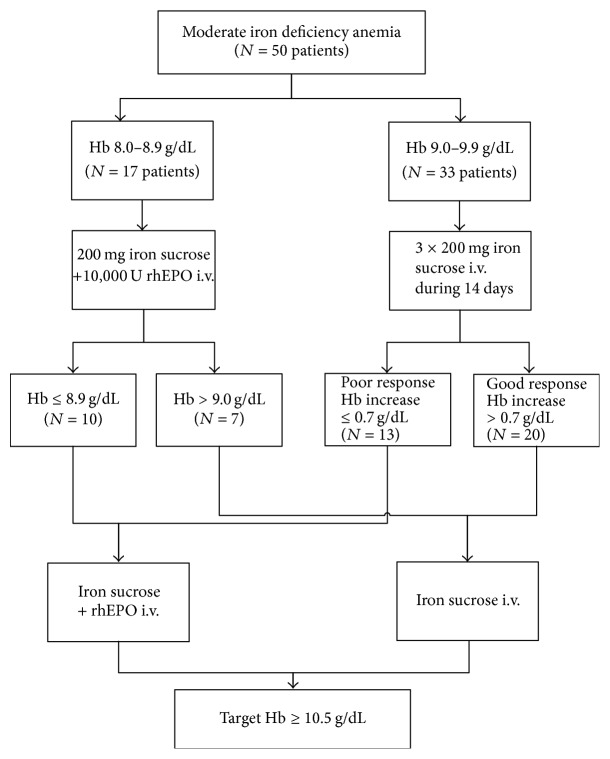
Therapy protocol.

According to hemoglobin level at the start of the therapy, the women were treated either with intravenous iron and rhEPO or with intravenous iron only twice weekly as described elsewhere [21, 26] (Figure 1).

Patients with an Hb level between 9.0 and 9.9 g/dl (33 patients) received 200 mg iron sucrose (VENOFER®, Vifor Int., St. Gallen, Switzerland) intravenously twice weekly (Figure 1). If response to therapy was poor (i.e., Hb increase <0.7 g/dl) after 2 weeks (13 patients), patients additionally received rhEPO (10,000 U EPREX®, Janssen-Cilag, Baar, Switzerland). On the basis of our previous experience we choose this cut-off for adequate primary response [21, 26, 27]. Patients with an Hb between 8.0 and 8.9 g/dl (17 patients) received iron sucrose (VENOFER) and rhEPO (EPREX) twice weekly from the start of therapy.

Sufficient overall response to therapy (the difference of baseline hemoglobin and that after therapy) was defined as Hb increase >1.0 g/dl. The maximum total iron dose was 1,600 mg; therefore therapy was stopped if the maximal iron sucrose dose was administered, or target Hb > 10.5 g/dl was achieved.

Side-effects (hypotensive and hypertensive response, allergic reaction, and thromboembolic complications) were registered.

### 2.4. Statistical Analysis

All statistical analyses were performed using the statistic program STATA 8 (Stata Corporation College Station, TX). *p* < 0.05 was considered statistically significant. Baseline characteristics were compared using Mann–Whitney and Chi-square tests when appropriate.

## 3. Results

**Figure 2:**
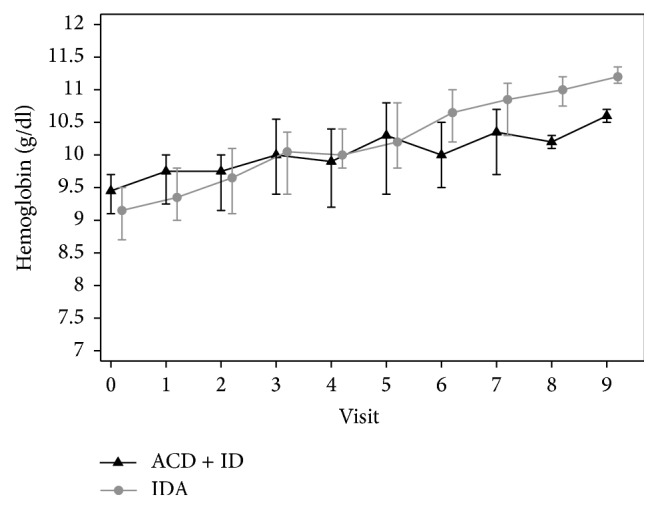
Course of Hb during therapy at the start and the end of the treatment.

The demographic characteristics are shown in Table 1. There were no differences between both groups with respect to maternal age, parity, gravidity, body mass index (BMI), and smoking. All patients showed stimulation of erythropoiesis as evidenced by an increase in reticulocytosis at day eight of therapy and showed an increase in hemoglobin and hematocrit at the end of therapy (*p* < 0.001) (Table 2) (Figure 2). The target Hb (≥10.5 g/dl) was achieved in 45/50 women (90%).

Anemia of chronic disease with reduced iron stores was observed in 24% (12/50) of which 59% (7/12) responded well to the treatment. Six of the 12 patients were treated with only iron sucrose, four patients were treated with iron sucrose and rhEPO in the second phase due to low response, and two patients received iron sucrose and rhEPO from the start of therapy.

An adequate response was ascertained in three of the six women treated only with iron sucrose (3/6; 50%) and in four of the six women treated with iron sucrose and rhEPO (4/6; 67%).

**Figure 3:**
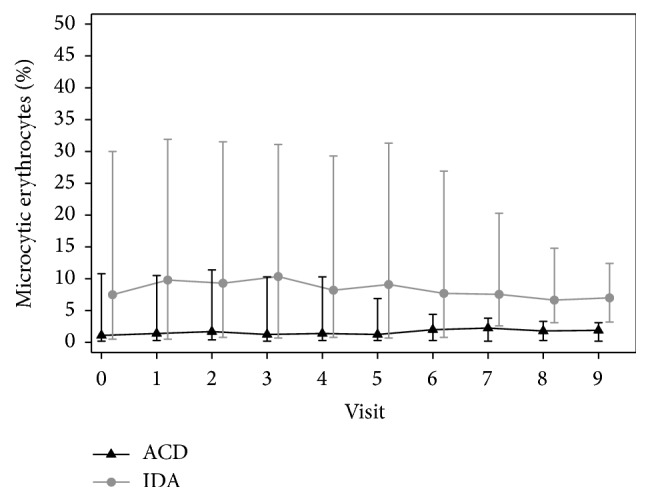
Respective MRC levels at the start and the end of the treatment.

**Figure 4:**
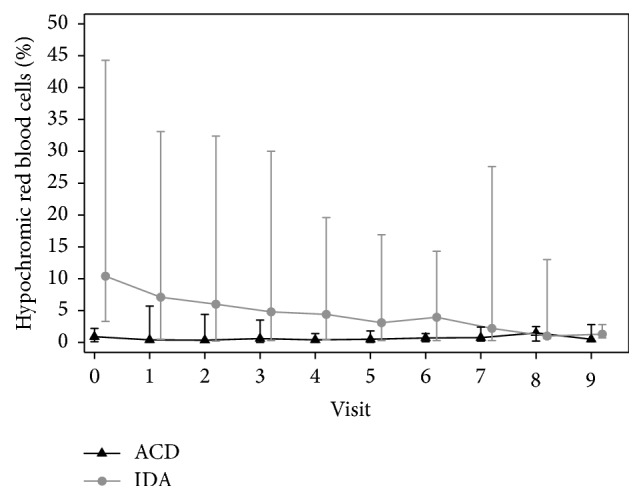
Respective HRC levels at the start and the end of the treatment.

Women with anemia of chronic disease with ID had a reduced ferritin prior to treatment (mean ferritin 11 ± 4.6 *μ*g/l and mean transferrin saturation 17.4 ± 12.5%) but had no signs of iron deficient erythropoiesis shown by normal levels of HRC (mean of HRC 1 ± 0.7%), RDW (mean of RDW 14.0 ± 1.5%), MRC (mean of MRC 2.2 ± 2.9%), and CHr (mean of CHr 30.6 ± 2.6 pg) at the start of the therapy (Figures 3 and 4). Serum EPO levels were insufficient for the grade of anemia (mean of serum EPO 42.1 ± 31.8 U/l prior to the treatment) and there was a statistically nonsignificant decrease of serum EPO at the end of the therapy compared to baseline EPO levels (*p* = 0.09). There was a statistically significant increase of Hb, HCT, and ferritin after the therapy in this group of women (*p* < 0.001). There was no statistically significant difference of MRC, HRC, CHr, and RDW after the therapy (*p* = 0.29; *p* = 0.94; *p* = 0.01; *p* = 0.09).

In the group of women with iron deficiency anemia, there was a statistically significant difference of all indicators of erythropoiesis (Hb, RBC, and HCT) and iron imbalance (CHr, HRC, RDW, and ferritin) after the therapy (*p* < 0.001) (Table 1).

Overall the mean total dose of iron sucrose per patient was 1,008 ± 390 mg (400–1,600 mg) and of rhEPO 17,000 ± 9,876 U (10,000–40,000 U). The maximal total iron sucrose (1,600 mg) was administered in 9/50 women; thereby 8/9 achieved the target hemoglobin. The mean duration of therapy was 3.0 ± 1.1 weeks (1–4.5 weeks).

None of the women needed antepartum or postpartum blood transfusion.

In all women, baseline vitamin B_12_, folic acid, and CRP were normal (the median of vitamin B_12_ was 162 ng/L, folic acid 6.8 *μ*g/L, and CRP 3.0 mg/L).

There were no significant differences in maternal outcome (Table 3). There were neither clinical nor laboratory signs of infection. There were no adverse events recorded to rhEPO and/or iron sucrose therapy. No hypotensive or hypertensive responses were seen during or after the therapy. There were no thromboembolic complications and no allergic reactions.

## 4. Comment

Our study investigated treatment of anemia of chronic disease with true iron deficiency in pregnant women. In our study group, twenty-four percent of the anemic patients suffered from anemia of chronic disease with true iron deficiency. In those patients, there were reduced iron stores and probably utilization as well as deficient erythropoiesis, but absolute iron incorporation in bone marrow was normal. Consequently, the appropriate anemic treatment is the administration of intravenous iron with rhEPO. Nevertheless, 50% of women (3/6) with anemia of chronic disease with ID responded well to intravenous iron, and 67% (4/6) responded well to the combination of intravenous iron and rhEPO.

It may be possible that iron deficiency in women with anemia of chronic disease is a latent iron deficiency, because of the significant difference of transferrin saturation prior to the treatment between those two groups (*p* < 0.001). Although depleted iron stores due to ferritin were determined in both groups, a higher level of free iron in the blood was observed in the group with normal HRC and CHr.

Anemia of chronic disease can be viewed as a spectrum of acute and chronic forms of anemia whose common pathophysiological denominator is their occurrence as a result of immune activation [28]. First, inflammatory mediators significantly impact on iron homeostasis, which results in iron limitation for erythropoiesis and subsequently development of anemia. The mainly liver-derived peptide hepcidin can be induced by inflammatory cytokines such as interleukin-1 and interleukin-6. This results in reduced uptake of dietary iron from enterocytes but also in impaired iron recycling by macrophages [29]. Second, inflammation negatively affects the formation and biological activity of the major red blood cell hormone erythropoietin. This is on the one hand due to reduced formation of the hormone caused by inhibitory cytokines and on the other hand linked to reduced erythropoietin receptor expression on erythroid progenitors and limited availability of iron [30, 31]. Third, inflammation inhibits the proliferation and differentiation of erythroid progenitor cells by multiple mechanisms [30]. The limitation of our study is the absence of measurement of these inflammatory mediators, hepcidin, erythroferrone, and soluble transferrin receptors. In our study, low serum EPO levels for the grade of anemia were observed in women with anemia of chronic disease.

Since the ACD is a direct consequence of an active immune-driven disease, its first-line therapy is treatment of the underlying condition. However, the subsequent therapeutic approach to ACD remains a matter of debate and clinical trials in future. In principle, there exist three traditional treatments for anemia: red blood cell transfusions, oral and intravenous iron administration, and injection of recombinant erythropoiesis-stimulating agents along with combinations thereof. The indications as well as the benefits and hazards of these specific treatments have been extensively reviewed. In patients with ACD + ID frequent administration of low doses of iron may be beneficial. Therefore, we used the dosage of 200 mg iron sucrose twice weekly. Prospective trials are required to optimize treatment regiments to ensure adequate efficiency of parenteral supplementation in different clinical settings. Therapy with rhEPO could be given from the start of therapy or as add-on therapy. Standard starting doses of rhEPO are 100–150 U/kg, administered subcutaneously three times a week. In agreement with previous studies, we administered 100–150 U/kg of rhEPO, although we administered rhEPO intravenously with iron sucrose twice weekly.

Iron deficiency anemia is characterized as anemia with depleted iron stores, elevated levels of hypochromic erythrocytes (>2.5%), decreased reticulocyte hemoglobin content (<28 pg), and elevated red blood cell distribution width (>15%). Those indicators were useful for the detection of iron imbalance and the monitoring of treatment as the proportion of hypochromic erythrocytes and the reticulocyte hemoglobin content decreased rapidly with adequate therapy (*p* < 0.001).

Our two-step treatment protocol regarding patients with Hb between 9.0 and 9.9 g/dl provided rapid differentiation of the patients with poor response to iron sucrose, early rhEPO supplementation to iron sucrose, and therefore antecedent achievement of target hemoglobin. However, Breymann et al. reported that ferric carboxymaltose may be a more appropriate option for rapid and effective anemia correction during late-stage pregnancy [32]. For some patients, a single dose of ferric carboxymaltose may correct iron deficiency anemia with no repeated administration required, thereby providing a more convenient option than other iron treatment and potentially increasing compliance [32]. The median time to achievement of target hemoglobin was 3.4 weeks in this study [32]. However, in our study, the mean time to achievement of target hemoglobin was 3.0 weeks.

The precise differential diagnosis between IDA, ACD, and a combination of both forms is of clinical importance because of differing treatment strategies. Currently, the lack of data from prospective clinical trials precludes definitive recommendations on diagnostic algorithms and prognostic indices [28]. These problems are aggravated by the lack of standardization in otherwise promising tests, such as measurement of soluble transferrin receptor and hepcidin. Further studies are necessary to investigate the prevalence, etiology, and outcome of anemia of chronic disease with ID in pregnancy. In our knowledge, there has been no study evaluating the prevalence of anemia of chronic disease with true iron deficiency in pregnancy. During pregnancy, often only the whole blood count, serum ferritin, and CRP are controlled. Because of frequent true iron deficiency in pregnant women with anemia of chronic disease, anemia of chronic disease in pregnancy is often falsely diagnosed as iron deficiency anemia.

## 5. Conclusions

Because of frequent true iron deficiency in pregnant women with anemia of chronic disease, anemia of chronic disease in pregnancy is often falsely diagnosed as iron deficiency anemia.

## Figures and Tables

**Table 1:** Demographic characteristics. Data expressed as median (range) or number (%).

	ACD + ID	IDA	*p* value
	(*n* = 12)	(*n* = 38)	
Maternal age (years)	27 (21–32)	26 (19–43)	0.7401
Gravidity (*n*)	2 (1–6)	2 (1–4)	0.4765
Parity (*n*)	1 (1–4)	2 (1–4)	0.5458
BMI (kg/m^2^)	20.7 (18.6–27.4)	22.7 (19.4–35.6)	0.5618
Smoking (*n*)	3/12 (25)	8/38 (21.1)	1.000
Gestational age at diagnosis of anemia (wk)	21.9 (19.7–37.1)	24 (21.1–38.6)	0.1439

**Table 2:** Hematological data, serum iron status, and serum EPO before and after treatment (^*∗*^*p* < 0.05, ^*∗∗*^*p* < 0.01, and ^*∗∗∗*^*p* < 0.001).

	ACD + ID			IDA		
	(*n* = 12)			(*n* = 38)		
	Mean ± SD		*p* value	Mean ± SD		*p* value
	Before	After		Before	After	
Hb (g/dl)	9.3 ± 0.5	10.8 ± 0.7^*∗∗∗*^	0.001	9.1 ± 0.5	11.1 ± 0.5^*∗∗∗*^	0.001
RBC (×10^6^/*µ*l)	3.20 ± 0.29	3.55 ± 0.21^*∗∗*^	0.002	3.67 ± 0.37	4.15 ± 0.37^*∗∗∗*^	0.001
HCT (%)	27.3 ± 1.6	31.7 ± 2.0^*∗∗∗*^	0.001	28.0 ± 1.8	33.4 ± 2.0^*∗∗∗*^	0.001
Reticulocytes (%)	2.32 ± 0.64	2.93 ± 0.56^*∗*^	0.036	2.42 ± 0.91	2.69 ± 0.92	0.247
MCV (fl)	85.7 ± 5.5	89.4 ± 4.2	0.074	76.9 ± 5.6	80.9 ± 5.1^*∗∗*^	0.002
MCH (pg)	29.3 ± 2.1	30.5 ± 1.2	0.089	24.9 ± 2.4	26.8 ± 2.1^*∗∗∗*^	0.001
MRC (%)	2.2 ± 2.9	1.2 ± 1.0	0.286	8.7 ± 7.2	9.8 ± 6.3	0.477
HRC (%)	1.0 ± 0.7	1.0 ± 1.1	0.948	12.4 ± 8.5	1.5 ± 0.9^*∗∗∗*^	0.001
CHr (pg)	30.6 ± 2.6	33.8 ± 1.0^*∗*^	0.014	25.7 ± 1.2	32.5 ± 0.6^*∗∗∗*^	0.001
RDW (%)	14.0 ± 1.5	15.8 ± 1.4	0.087	15.2 ± 0.7	19.8 ± 0.5^*∗∗∗*^	0.001
Iron (*µ*mol/l)	12.6 ± 7.3	19.0 ± 4.4	0.094	7.2 ± 5.3	15.0 ± 5.5^*∗∗∗*^	0.001
Transferrin (*μ*mol/l)	40.1 ± 10.5	34.2 ± 5.6	0.266	50.1 ± 11.3	42.9 ± 9.8^*∗*^	0.030
Ferritin (*µ*g/l)	11.0 ± 4.6	177.5 ± 69.1^*∗∗∗*^	0.001	6.2 ± 2.8	126.0 ± 72.5^*∗∗∗*^	0.001
Transferrin saturation (%)	17.4 ± 12.5	27.7 ± 5.1	0.106	8.2 ± 7.1	18.7 ± 8.5^*∗∗∗*^	0.001
EPO (U/l)	42.1 ± 31.8	21.1 ± 5.9	0.087	94.7 ± 52.3	37.9 ± 22.1^*∗∗∗*^	0.001

**Table 3:** Maternal outcome. Data expressed as mean ± s.d. (range) or number (%).

	ACD + ID	IDA	*p* value
Fasting glucose (mmol/l)	4.3 ± 0.4 (3.4–4.7)	4.4 ± 0.4 (3.8–5.6)	0.4539
GDM (*n*)	0/12	2/38	1.000
Preeclampsia (*n*)	1/12	2/38	1.000
IUGR (*n*)	0/12	2/38	1.000
Weight gain (kg)	18.3 ± 7.2 (5.9–30)	15.8 ± 6.2 (5–31)	0.2471
Delivery modus (%)			0.721
Nonoperative vaginal delivery	5/12 (41.7)	20/38 (52.6)	
Operative vaginal delivery	2/12 (16.6)	4/38 (10.5)	
Cesarean section	5/12 (41.7)	14/38 (36.9)	
Gestational age at delivery (wk)	39 ± 2 (35–42)	40 ± 2 (34–42)	0.1376
Birth weight (g)	3318 ± 550 (2170–4020)	3203 ± 514 (1610–3960)	0.5094
